# Sensorimotor organization of a sustained involuntary movement

**DOI:** 10.3389/fnbeh.2015.00185

**Published:** 2015-07-28

**Authors:** Jack De Havas, Arko Ghosh, Hiroaki Gomi, Patrick Haggard

**Affiliations:** ^1^Action and Body, Institute of Cognitive Neuroscience, University CollegeLondon, UK; ^2^Institute of Neuroinformatics, University of Zurich and ETH ZurichZurich, Switzerland; ^3^Neuroscience Center Zurich, University of Zurich and ETH ZurichZurich, Switzerland; ^4^NTT Communication Science Laboratories, Nippon Telegraph and Telephone CorporationAtsugi, Japan

**Keywords:** involuntary contraction, motor control, efference copy, involuntary movement, sensory feedback

## Abstract

Involuntary movements share much of the motor control circuitry used for voluntary movement, yet the two can be easily distinguished. The Kohnstamm phenomenon (where a sustained, hard push produces subsequent involuntary arm raising) is a useful experimental model for exploring differences between voluntary and involuntary movement. Both central and peripheral accounts have been proposed, but little is known regarding how the putative Kohnstamm generator responds to afferent input. We addressed this by obstructing the involuntary upward movement of the arm. Obstruction prevented the rising EMG pattern that characterizes the Kohnstamm. Importantly, once the obstruction was removed, the EMG signal resumed its former increase, suggesting a generator that persists despite peripheral input. When only one arm was obstructed during bilateral involuntary movements, only the EMG signal from the obstructed arm showed the effect. Upon release of the obstacle, the obstructed arm reached the same position and EMG level as the unobstructed arm. Comparison to matched voluntary movements revealed a preserved stretch response when a Kohnstamm movement first contacts an obstacle, and also an overestimation of the perceived contact force. Our findings support a hybrid central and peripheral account of the Kohnstamm phenomenon. The strange subjective experience of this involuntary movement is consistent with the view that movement awareness depends strongly on efference copies, but that the Kohnstamm generator does not produces efference copies.

## Introduction

Ludwig Wittgenstein famously asked “What is left over if I subtract the fact that my arm goes up from the fact that I raise my arm?”(Wittgenstein, 2009). The voluntary command to raise one's arm is so tightly coupled to the feeling of the arm rising that the two often appear indistinguishable. However, this familiar phenomenology belies the complexity of the motor control hierarchy recruited in even simple voluntary actions. Multiple involuntary processes are required to translate a high level goal into the specific patterns of muscle activity that characterize the initiation, maintenance and cessation of movement (Scepkowski and Cronin-Golomb, 2003; Fowler et al., 2008; Scott, 2012). Yet the detailed implementation of a voluntary action remains outside conscious awareness: one feels *entirely* in control of a process which, in fact, is merely *initiated* voluntarily. In contrast, when the cause of body movement is external, as when one's arm is lifted by another person, the event is unambiguously felt as external. Most models of action control suggest that the critical difference between a voluntary action and a passive movement is the presence or absence respectively of an efference copy of the motor command. When sensory information from the moving arm can be canceled by an efference copy, the action is perceived as voluntary (Blakemore et al., 1998).

Another established distinction in motor control contrasts voluntary movements to reflexes. Reflexes are stereotyped, rapid responses to a specific afferent signal (Kimura et al., 2006). Although not initiated voluntarily, they are modulated by task and voluntary set (Overduin et al., 2012). The awareness of reflexive movements has rarely been studied. Isolating the motor commands of these movements, and determining how they contribute to action awareness is difficult, because of their rapid onset, short duration, and close interaction with afferent signals (Ghosh and Haggard, 2014).

Here, we use the Kohnstamm phenomenon (Kohnstamm, 1915) as a convenient experimental model for comparing reflex and voluntary movement, and thus for isolating the specific elements of motor awareness that depend on voluntary control. In the Kohnstamm phenomenon, a strong, sustained, isometric muscle contraction produces, upon relaxation, a sustained aftercontraction in the same muscle. In a classic, party-trick version, participants press outwards with the back of the hands against a doorframe for around 1 min. Stepping forward away from the doorframe and relaxing the arm muscles is followed by the arms involuntary rising, or “levitating.” The movement differs from other postural reflexes such as stretch in two ways: it is slow and prolonged, and it is largely confined to a single muscle (But see Duclos et al., 2004). Crucially, while the involuntary movement produced by the aftercontraction falls within the same temporal and force range as voluntary movement, it feels subjectively very different. The movement is surprising (Forbes et al., 1926; Craske and Craske, 1985), with the arm feeling lighter than normal (Kohnstamm, 1915; Cratty and Duffy, 1969; Craske and Craske, 1985; Gurfinkel et al., 1989; Hagbarth and Nordin, 1998), as if it is floating (Salmon, 1914; Craske and Craske, 1985), either of its own accord (Craske and Craske, 1985) or via some “hidden force” (Kohnstamm, 1915).

The Kohnstamm phenomenon has been interpreted as a result of neural adaptation within a postural control system (Gurfinkel et al., 1989; Ghafouri et al., 1998; Duclos et al., 2004, 2007; Parkinson and McDonagh, 2006). The postural control system is thought to maintain a reference value of motor activity against external perturbation or voluntary movement (Massion, 1992; Adamson and McDonagh, 2004). This implies an ability to adjust to transient afferent input, before returning to the desired level of motor output. In normal circumstances, many movements include both a postural and a voluntary goal-directed component. These two components are controlled by quite different mechanisms, but may nevertheless be experienced as a single event (Gurfinkel et al., 1989; Ghafouri et al., 1998; Ghosh and Haggard, 2014). In contrast, in the Kohnstamm aftercontraction, a postural component is experienced in isolation, without any voluntary component.

The mechanisms behind the Kohnstamm phenomenon are poorly understood. On one, peripheralist, view, the Kohnstamm generator is driven by a sustained afferent discharge (Gregory et al., 1988; Hagbarth and Nordin, 1998; Duclos et al., 2004). Consistent with this view, microneurographic recordings showed increased spindle firing rates following isometric contractions (Ribot-Ciscar et al., 1991, 1998; Wilson et al., 1995). Muscle thixotropy may result in fusimotor fibers continuing to stretch the spindles after the end of voluntary contraction (Hagbarth and Nordin, 1998). This would in turn generate an aftercontraction via spinal or supraspinal reflexes (Hutton et al., 1973; Smith et al., 1974; Durkovic, 1976; Gregory et al., 1986; Hagbarth and Nordin, 1998). Indeed, involuntary movement similar to the Kohnstamm can be generated from sustained mechanical vibration applied to a single muscle (Gilhodes et al., 1992; Duclos et al., 2007). Further, vibration-induced and Kohnstamm movements produce a similar pattern of brain activity (Duclos et al., 2007).

Alternatively, the Kohnstamm phenomenon may be caused by a central adaptation. It has been proposed that the Kohnstamm generator is a persistence of the inducing voluntary contraction (Salmon, 1914, 1916), possibly reflecting changes in the excitatory state of the motor cortex (Sapirstein et al., 1936, 1938). Indeed, it has been reported that it is possible to induce the Kohnstamm phenomenon via sustained motor mental imagery (Craske and Craske, 1986). Recent neuroimaging work supports the central adaptation account. Aftercontractions were associated with widespread cortical activations resembling those seen during voluntary movement (Duclos et al., 2007; Parkinson et al., 2009). Further, applying transcortical magnetic stimulation to the motor cortex during the aftercontraction induces a silent period in the contracting deltoid muscle (Ghosh et al., 2014). The silent period did not differ in terms of latency or duration from that obtained during a matched voluntary movement. This suggests that that the motor cortex can be considered part of the Kohnstamm generator.

The Kohnstamm generator may therefore be activated by either peripheral, or central sources, or a hybrid of both. Establishing whether the Kohnstamm generator is altered by sensory inputs may clarify this question. Specifically, a purely central, feedforward generator should be unaffected by peripheral sensory input. A purely peripheral mechanism could, potentially, be entirely reset by a novel peripheral input, stopping the Kohnstamm contraction entirely. Here, we obstruct the rising arm to determine if sensorimotor feedback forms part of the Kohnstamm control circuitry. Because this obstruction has clear perceptual correlates, it can be used to quantify the subjective experience of the aftercontraction. The response to a physical obstruction has proved important in understanding neural mechanisms of central pattern generation (CPG), as in control of stepping behavior (Duysens and Van de Crommert, 1998; McVea and Pearson, 2006, 2007). However, this approach has rarely been applied to involuntary movements.

Visual and proprioceptive input from the other arm can affect aftercontractions under specific conditions (Gilhodes et al., 1992; Brun et al., 2015). However, only two studies have previously investigated the interaction between aftercontractions and sensory input from physical obstruction. Forbes et al. (1926) reported that contacting an obstacle does not abolish the aftercontraction. Adamson and McDonagh (2004) reported that blocking the rising arm resulted in a constant EMG whose amplitude was proportional to the arm angle at the time of the block. However, these studies did not address how this sensory information regarding obstruction might affect the Kohnstamm generator. Specifically, they did not investigate how the muscle activity changed over time in response to contacting the obstacle, relative to a matched, unobstructed aftercontraction. Further, they did not attempt to quantify the subjective experience of encountering obstruction during Kohnstamm aftercontraction. Finally, they did not address whether obstruction had a lasting or transient effect on muscle activity, nor whether the effects were unilateral or bilateral. Thus, several questions remain about the sensorimotor organization of the Kohnstamm aftercontraction, and in particular about the effects of sensory input from obstruction.

We have therefore conducted two experiments to address the following research questions: (1) Does the Kohnstamm generator rely solely on central feedforward control or is it modulated sensorimotor feedback? (2) Does one Kohnstamm generator drive aftercontractions in both sides of the body, or does a separate generator exist for each side (3) Is the sensory response of the muscle the same as during voluntary movement? (4) Are the forces from movements produced by the generator perceived differently to voluntary movements? Experiment 1 assessed the effects of random and unexpected obstruction of a unilateral Kohnstamm on EMG. Perception of force relative to voluntary and passive movements was explicitly reported. Experiment 2 assessed the effects of obstructing one arm during a bilateral Kohnstamm and then removing this obstacle. Perception of contact force, relative to voluntary movements, was again investigated, this time via an implicit force matching task.

## Experiment 1

### Methods

#### Equipment

The setup is schematically shown in **Figure 2**. Electromyography (EMG) was recorded from bipolar, surface electrodes placed over the middle of the lateral deltoid, parallel to the orientation of the muscle fibers. The electrodes were connected to a 1902 amplifier (Cambridge electronic design), which was controlled via custom Labview scripts (sample rate = 2000 Hz, gain = 1000, 50 Hz notch filter). Pilot studies showed that small changes in posture across trials could lead to large differences in the arm position during aftercontraction. To ensure that the arm was completely stopped on all obstruction trials, a rigid steel rod (length = 20 cm, diameter 1 cm) instrumented with strain gauges was used to obstruct movements. The gauges were connected to amplifiers (low pass filter = 10 kHz, high pass filter = DC, 50 Hz notch filter). However, the strain gauges were calibrated offline, so that the force exerted at a known location on the rod could be calculated. A camera was used to continuously record the force rod so that the position of every arm contact could be coded. Kinematics were recorded via a second video camera (60 fps) and two LEDs attached to the participant's arm at the shoulder (fixed point) and upper arm (moving point). Participants wore goggles to limit visual input and wrist and elbow splints to ensure their arms stayed straight during shoulder abductions.

#### Participants

In total 23 participants (14 female, mean age = 23.8 years old) were recruited for the experiment. However, seven participants were not included in the final analysis because they either: (1) voluntarily withdraw from the experiment (*n* = 1), (2) did not display an aftercontraction (*n* = 3), or (3) displayed an aftercontraction that did not produce sufficient arm movement to contact the obstacle (*n* = 3). This left 16 participants (9 female, mean age = 23.6 years old) in the final analysis. Experiments were undertaken with the understanding and written consent of each participant in accordance with the Code of Ethics of the World Medical Association (Declaration of Helsinki), and with local ethical committee approval.

### Procedure

**Figure 1:**
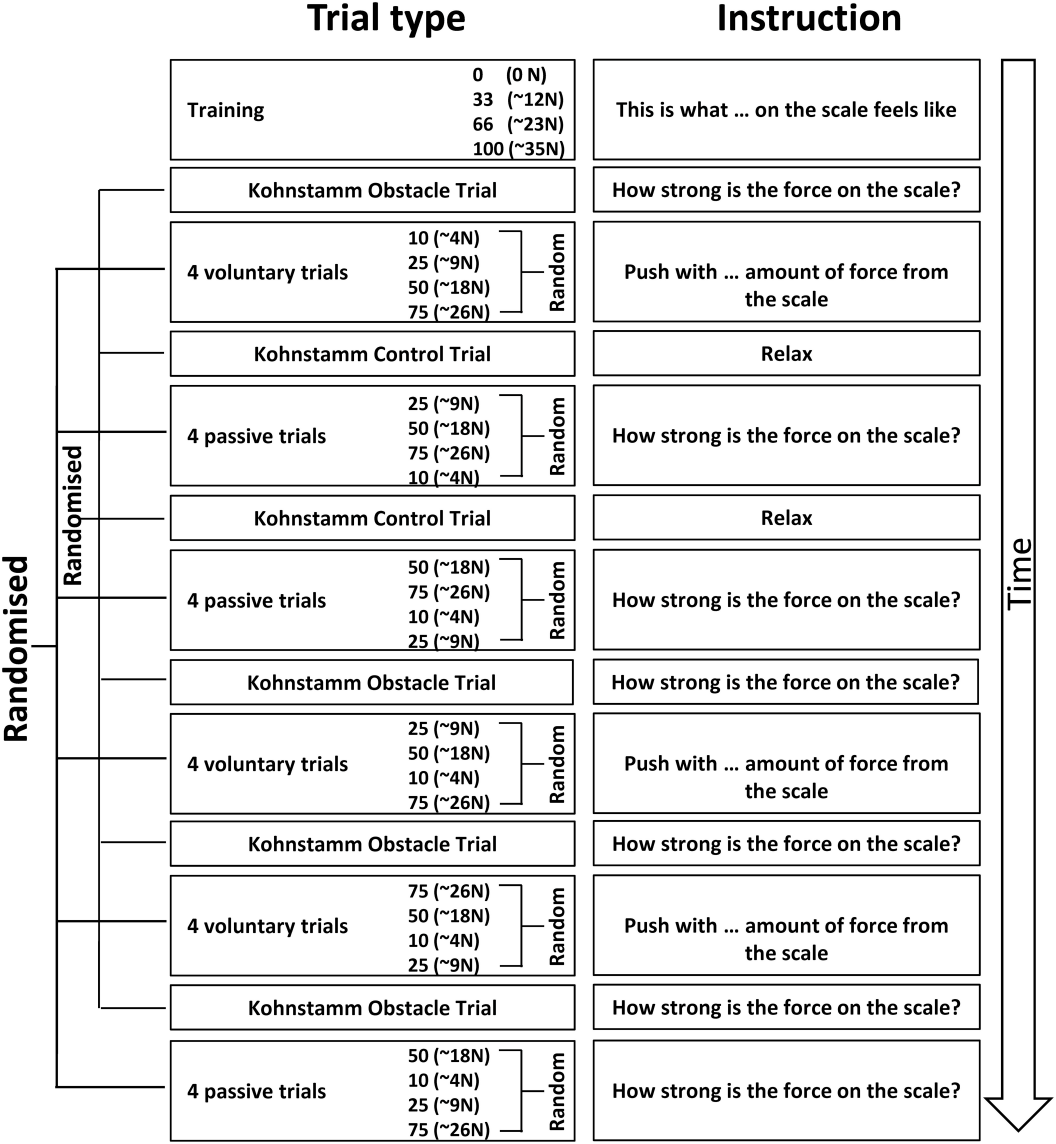
**A schematic of Experiment 1 showing the order in which the trials were experienced and the specific instructions given to the participants**. Training was always completed first, followed by a Kohnstamm trial. The order of Kohnstamm trial types was randomized and counterbalanced across participants. Next were blocks of either Voluntary or Passive Movement trials, which were separately randomized and counterbalanced. Within each block of Voluntary or Passive trials there was always one trial at each force level. The specific order was randomized.

Before the experiment began, participants were instructed to perform a brief maximal isometric voluntary contraction (MVC) of the lateral deltoid muscle by pushing outwards against a wall for 5 s. They were told that from that point on they should aim to reproduce approximately 70% MVC for all subsequent isometric contractions. In line with previous studies of the Kohnstamm phenomenon (Craske and Craske, 1985; Duclos et al., 2007; Ghosh et al., 2014), we chose to use this subjective criterion of induction force to maximize the likelihood of getting reliable aftercontractions. EMG was monitored online to ensure participants were complying with this level of effort throughout the remainder of the task. A schematic of the entire experiment is shown in Figure 1. Participants were familiarized with a scale for subjective rating of forces. Participants were told that throughout the experiment they would be using a linear scale from 0 to 100 to report the amount of force they were experiencing. The experimenter then demonstrated the meaning of the numerical scale by passively lifting the participant's arm against the force rod in order to achieve an announced level of force. Thus, participants learned that an experienced force of 12 N was labeled 33 on the scale, 23 N was labeled 66, and 35 N corresponded to 100 on the scale. They were further told that a value of 0 corresponded to no force at all. This procedure aimed to instruct participants in rating a set of equispaced force levels. In practice, there were small variations, because the reading from the strain gauges depended not only on the actual force applied, but also on the location of the contact along the rod. Thus, the actual force applied during instruction was known only after subsequent calibration taking the position of force application into account. See Supplementary Materials for diagram of apparatus (Supplementary Figure 1).

**Figure 2:**
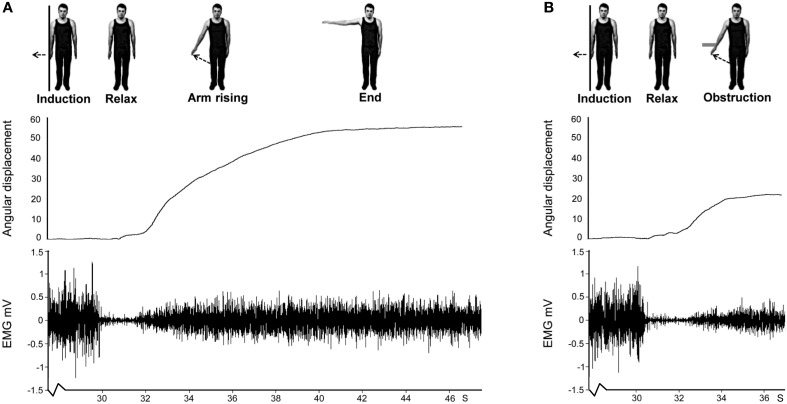
**Schematic for Experiment 1 showing arm displacement and EMG from a representative no obstruction (A) and obstruction (B) trial**. Note that only the last ~2 s of the 30 s isometric induction contraction are shown for both trials. This is followed by relaxation of the muscle which lasted ~1.5 s in this participant. The aftercontraction then began, accompanied by involuntary movement. In the no obstruction trial **(A)** the arm rose unimpeded. In the obstruction trial **(B)** an obstacle stopped the arm at ~20°.

At the start of each Kohnstamm trial, participants were instructed to stand upright with their palms facing inwards, and their arms relaxed and by their sides. The only object that participants could see was an LED placed at eye level on the opposite wall. The LED was controlled by the experimenter, and was used to trigger the different phases of each trial. The first LED onset signaled participants to begin a continuous, unimanual, isometric contraction of the lateral deltoid at 70% MVC. After 30 s the LED signaled participants to stop pushing, step forward and relax. An aftercontraction of the lateral deltoid then occurred causing the arm to abduct. During “No Obstruction” trials (Figure 2A) the arm was allowed to rise unimpeded and participants were simply instructed to stay relaxed and let the arm rise and fall whenever it felt natural to do so. In the obstruction trials (Figure 2B) the arm was blocked by the instrumented rod after around 20° of abduction. After ~1 s of contact, a further LED signal instructed participants to report the amount of force they were experiencing using the 0–100 scale. Participants were naïve to whether the obstacle was going to be present or not in any trial, and trial order was randomized.

Kohnstamm trials alternated between the left and right arm. Participants completed 6–9 trials (Mn = 7.44, *SD* = 1.26), comprising at least two no obstruction trials, and at least four obstruction trials (Figure 1). The number of trials could vary because sometimes the arm did not abduct far enough to reach the obstacle. In these instances the trials were repeated. After every Kohnstamm there was a 3 min rest. Following rest, participants engaged in blocks of four Voluntary and Passive trials (in randomized order). These trials were systematically alternated with Kohnstamm conditions, rather than tested in a separate block. We reasoned that alternation would help to prevent long-lasting motor post-effects (Hutton et al., 1987; Duclos et al., 2004). Voluntary trials consisted of the experimenter giving the participant a number on the force scale. The numbers were drawn from four distributions centered on 10, 25, 50, and 75 (one from each per block). Participants then had to abduct their arm and push against the force rod with the amount of force they thought corresponded to the number they had been given, based on their previous learning of the scale. The experimenter recorded when the participant felt they had generated the correct amount of force with a button press. On Passive trials the experimenter lifted the participant's arm against the force rod, attempting to achieve one of four pre-set levels of force (~4, 9, 18, and 26 N), designed to correspond to ratings of 10, 25, 50, and 75, respectively on the previously-learned numerical scale. As before, the experimenter's passive force generation could only be approximately accurate, because the experimenter monitored a raw force signal, and the actual force was known only after offline calibration, taking into account the position of the participant's hand along the force rod. The analysis used the actual, calibrated force levels for each participant. Once the experimenter achieved the target force level, the LED was switched on, and participants verbally reported the current force level, as a rating between 0 and 100. All participants completed three blocks of Voluntary trials and three blocks of Passive trials (counterbalanced). The experiment lasted approximately 2 h.

### Analysis

**Figure 3:**
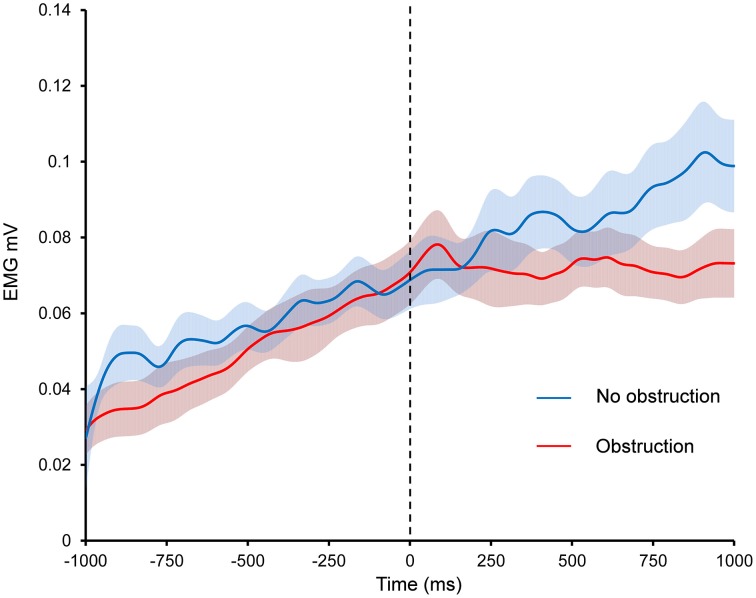
**The effect of obstruction on EMG during Kohnstamm**. Dashed line indicates time of obstruction in obstruction condition and time when obstruction would have occurred in the no obstruction condition. Error bars show SEM.

Kinematics analysis was performed by determining the angle between the horizontal and two LEDs, placed on the shoulder and forearm using ImageJ (Schneider et al., 2012). EMG was band pass filtered (10–500 Hz) and rectified. For display purposes the rectified EMG was smoothed with a 4 Hz low pass filter (Figure 3). On obstruction trials, the point in time when the participant made contact with the obstacle was determined from the strain gauges mounted in the obstacle. Four 250 ms bins were created either side of this time point. The mean EMG in each bin across all obstruction trials was then calculated for every participant. Next, using the kinematics data, the angular displacement for the obstacle on every obstruction trial was determined individually for each participant. The mean was then calculated and this was taken as the point in space and time where the obstacle would have appeared on the no obstruction trials. This was performed to account for small variations in the position of the obstacle relative to the participant across trials. Although the obstacle was in a fixed location, minor postural changes meant that the precise angle of the arm when contacting the obstacle could vary across trials. Again four 250 ms bins were created either side of this time point. The mean EMG from each bin across all no obstruction trials was then calculated for every participant. Because the EMG generally increased linearly during this time, a linear trend was fitted to quantify the change in EMG over time, using the standard coefficients −3, −1, 1, 3 for the four successive bins prior to the contact, and again for the four bins after contact. Contrast coefficients were calculated by multiplying mean EMG signal in the four 250 ms bins by the standard coefficients. The average EMG trend value could then be calculated for each participant in the two 1 s windows of interest in each of the two conditions (see Supplementary Table 1). A 2 × 2 within subjects ANOVA with the variables Time (before contact point vs. after contact point) and Condition (obstruction vs. no obstruction) was then performed on the trend values to assess if contact with the obstacle altered the EMG pattern. Any trial where the participant's arm did not reach the obstacle (obstruction trials), or the corresponding point in no obstruction trials, was excluded from the above analysis.

To calculate the force between the participant's arm and the obstacle, we took into account the position along the steel rod that the participant's forearm made contact on every trial. An analysis window of 500 ms was selected and the mean force within this time-bin was calculated for every trial. In the Kohnstamm and Passive conditions this bin was directly after the onset of the button press/light which instructed participants to report their force ratings. In the Voluntary condition the 500 ms bin was centered on the onset of the button press/light onset to ensure that the analysis corresponded to the point in time where participants felt they had achieved the correct level of force. For every trial the subjective rating of force was divided by the actual force, to produce a value indicating the perceptual intensity per unit of physical force. These values were then averaged across conditions for each participant. Statistical analysis was then performed via a One-Way within subjects ANOVA.

### Results

#### Obstruction reduces linear trend of EMG relative to an unobstructed Kohnstamm

As can be seen from Figure 3, contact with the obstacle reduced the linear trend of EMG activity relative to an unobstructed Kohnstamm. The ANOVA based on linear trend analysis showed a significant main effect of Time [*F*_(1, 15)_ = 6.5, *p* = 0.02], a significant main effect of Condition [*F*_(1, 15)_ = 5.75, *p* = 0.03] and significant Time × Condition interaction [*F*_(1, 15)_ = 8.85, *p* = 0.01]. *Post hoc t*-tests showed a significant decrease in the linear trend of EMG during the 1000 ms after contact with the obstacle, relative to before the obstacle, in the obstruction condition only [*t*_(15)_ = 3.67, *p* = 0.002]. There was no significant change in the linear trend of the EMG in the no obstruction condition [*t*_(15)_ = −0.39, *p* = 0.7]. Trend values can be found in Supplementary Table 1.

#### Kohnstamm forces are rated as higher than passive and voluntary forces

In the Kohnstamm condition, the mean subjective rating of force divided by actual force was 20.67 (*SD* = 20.68), whereas in the Passive condition it was 3.64 (*SD* = 1.7) and in the Voluntary condition it was 3.81 (*SD* = 2.12; Table 1). A significant effect of condition was found [*F*_(1, 15)_ = 10.5, *p* = 0.005, Greenhouse-Geisser corrected]. *Post hoc t*-tests revealed that experienced force was significantly higher in the Kohnstamm condition compared to the Passive condition [*t*_(15)_ = 3.33, *p* < 0.05, Bonferroni corrected] and Voluntary condition [*t*_(15)_ = 3.17, *p* < 0.05, Bonferroni corrected]. There was no significant difference between the Passive and Voluntary conditions.

**Table 1:** **Rating of force divided by actual force for Kohnstamm, Passive, and Voluntary movements**.

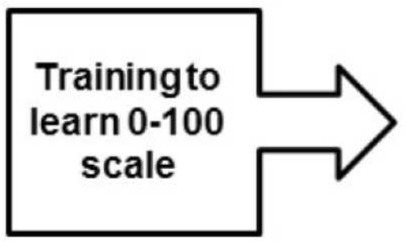	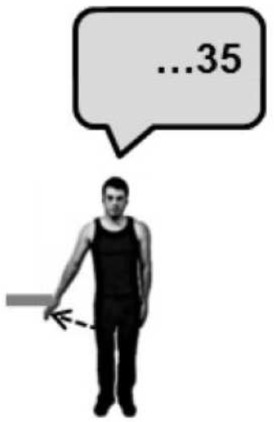	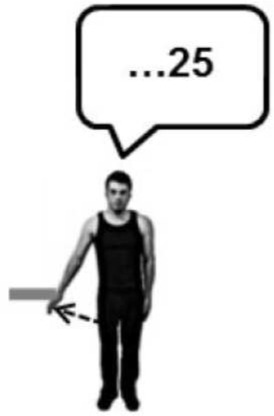	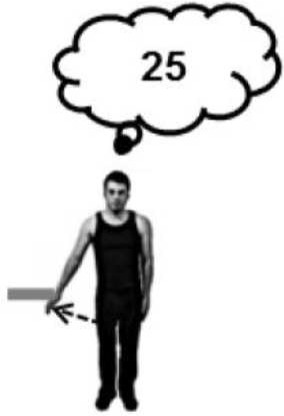
**Rating/force**	**Kohnstamm**	**Passive**	**Voluntary**
Mean	20.67	3.64	3.81
SD	20.68	1.70	2.12

### Discussion

Obstructing a Kohnstamm aftercontraction with an obstacle produced a clear plateau in the agonist EMG signal. A single EMG trace from a single participant in an earlier paper shows, but does not quantify, a similar phenomenon (Forbes et al., 1926). Later work examined the effect of stopping the Kohnstamm at different arm angles (Adamson and McDonagh, 2004), but (a) did not include an unobstructed condition, and (b) focused on the EMG level at each angle of arm abduction, rather than how contacting an obstacle affects EMG in the time domain. By comparing obstruction and no obstruction trials, we showed for the first time that it is the obstruction, and associated afferent input, that causes the change in EMG signal. However, two important questions remain. First, is this influence permanent, or does it end when the obstacle is removed. Second, how does peripheral sensory information interact with the Kohnstamm generator? These questions are addressed in Experiment 2.

Kohnstamm forces were rated as being subjectively stronger than voluntary and passive forces applied to the same area of the forearm. Overestimation of force during Kohnstamm could reflect lack of an efference copy to cancel against the sensory consequences of the action (Blakemore et al., 1998). Efference copy is often invoked to explain the relative underestimation of voluntary compared to passive forces (Shergill et al., 2003). Interestingly, however, we did not reproduce this result in our dataset. Thus a lack of efference cannot fully explain the results of Experiment 1 (see General Discussion for a consideration of involuntary and passive movements). However, the range of forces in the Kohnstamm condition could not easily be matched to the other conditions. Therefore, the subjective perception results from Experiment 1 remain rather tentative. The explicit reporting of force could also encourage participants to respond to the overall “strangeness” of the Kohnstamm, meaning the overestimation of force could be postdictive. As such, an implicit force reproduction task was used in Experiment 2.

## Experiment 2

### Methods

#### Equipment

EMG was recorded in the same manner as Experiment 1 simultaneously from the left and right lateral deltoid muscles. An adjustable doorframe was built using two vertical metal poles, positioned such that each participant could comfortably stand between them and push outwards with both arms 10° abducted. Unlike Experiment 1, in this experiment it was necessary to have an obstacle that could be applied randomly to each arm in an alternate fashion. Thus the fixed obstacle previously used was inappropriate. Obstacle contact force was recorded using a strain gauge (Mecmesin Advanced Force Gauge) fitted with a flat circular metal disc (diameter = 2 cm). The strain gauge was placed inside a wooden casing that could be braced against the experimenter who stood against a solid surface (see Supplementary Figure 1). Data was acquired in the same manner as Experiment 1. A webcam was used to record the session and participants were again fitted with LEDs. Participants also wore earplugs to avoid any sound cues from the experimenter or apparatus regarding the repositioning of the obstacle from one arm to the other.

#### Participants

Inclusion criteria were the same as for Experiment 1. In total 18 participants (7 female, mean age = 24.5 years old) were recruited. Of these, six were excluded from the final analysis for the following reasons: (1) voluntarily withdrew from the experiment (*n* = 1), (2) did not display an aftercontraction (*n* = 1), never displayed an aftercontraction large enough to produce 20° of angular displacement (*n* = 4). This final exclusion criterion was necessary as the unobstructed arm needed to be capable of rising above the point in space where the obstacle was applied (~15°) for the analysis to be meaningful. This left 12 participants in the final analysis (3 female, mean age = 25.2 years old). None of these participants had participated in Experiment 1.

### Procedure

**Figure 4:**
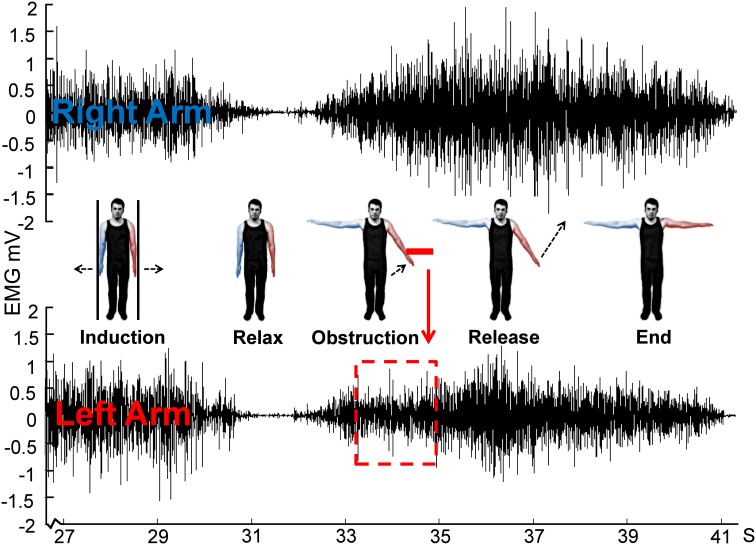
**Schematic for Experiment 2 showing EMG of obstructed left arm and unobstructed right arm from a single representative trial**. Note that only the last ~3 s of the 30 s isometric induction contraction is shown.

The participant's MVC was established as before, and they were once again instructed to push with 70% MVC to induce a Kohnstamm effect. Kohnstamm trials were the same as in Experiment 1, with the important difference that this time participants pushed outwards with *both* arms. Participants were simply instructed to allow any arm movements that might follow the induction process. As the aftercontraction began, the experimenter obstructed one arm after ~15° of angular displacement using the braced strain gauge applied to the dorsal forearm just above the wrist. The other arm was free to rise unobstructed (Figure 4). Based on pilot experiments, it was hypothesized that removing the obstacle after a short duration would result in the arm continuing to rise involuntarily. This would require an increase in EMG. The obstacle was thus removed after ~2 s allowing the obstructed arm to rise. Participants knew that one arm would be obstructed on each trial, but were unaware which it would be. They were instructed to remember the force with which their arm had hit the obstacle. Once both arms had ceased moving, participants were told to bring their arms back to the start position and relax. The experimenter then verified that the arm was completely stationary and all signs of the aftercontraction had ended. After 1 min participants were told to reproduce the force with which they had just hit the obstacle via a voluntary movement. Unlike Experiment 1, here participants had not been told about any subjective force scale. The obstacle was in the same position as during the aftercontraction.

After a 2 min rest, participants then completed a voluntary trial. On these trials participants were instructed to raise both their arms at the same speed as during the Kohnstamm trials. Once again the experimenter would obstruct one of the arms for 2 s at ~15° of angular displacement and then release it. The other arm was free to rise unobstructed. Again participants were naïve to which arm would be obstructed. Once both arms had stopped moving the experimenter instructed the participant to bring them down. As before, they were instructed to remember the force with which they hit the obstacle and after 1 min reproduce that force.

Participants completed 4–6 Kohnstamm trials (Mn = 5.08, *SD* = 0.67) and a matched number of Voluntary trials. Trial number varied because sometimes it was necessary to repeat a trial where the arms did not rise past ~15° of angular displacement. The obstructed arm was independently randomized for the Kohnstamm and Voluntary trials to minimize any expectation on the part of the participant. During post-test questioning all participants stated that they could not guess in advance which arm would be obstructed. The experiment lasted ~2 h.

### Analysis

EMG analysis centered on the contact with the obstacle, as Experiment 1. The detection of contact with the obstacle was based on the signal from the strain gauge. Statistical analysis was broadly as in Experiment 1. The factor of Time (before contact point vs. after contact point) was included in the ANOVA. We also included a factor of Arm to distinguish between the arm that did contact the obstacle on each trial, and the other arm that did not.

**Figure 5:**
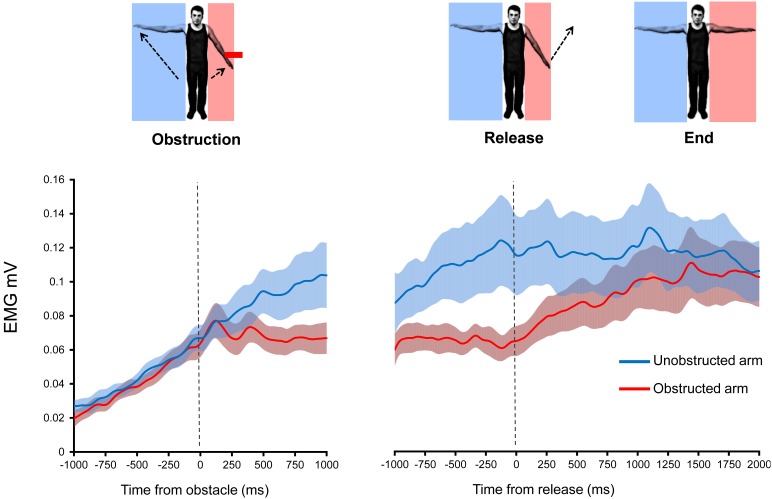
**Effects of introduction and removal of an obstacle on both the unobstructed and obstructed arm during bilateral Kohnstamm**. Error bars show SEM.

Unlike Experiment 1, the obstacle was removed after ~2 s, and the arm released. The effects of releasing were investigated in the same way as the effects of contacting the obstacle: resampling of EMG into time bins, linear trend analysis and ANOVA were performed as for the onset of contact. Smoothing (4 Hz) was performed as before only for the purposes of displaying the data (Figure 5). In the case of the release-locked analysis, data is shown for 2 s after the release (statistical analysis performed on 1 s, split into four bins). The additional 1 s of data was included to determine whether the EMG in the obstructed arm reached the same level as the unobstructed arm. A direct comparison via *t*-test was performed on the final 250 ms bin across both arms.

We specifically investigated EMG transients just after contact with the obstacle to measure possible stretch reflexes. An analysis window of 60–160 ms post-contact was used, as this is thought to correspond to long loop transcortical reflexes (Conrad and Meyer-Lohmann, 1980; Matthews, 1991). Since EMG increases during the Kohnstamm, any reflex would be superimposed on an underlying Kohnstamm pattern. We therefore used a special procedure to estimate reflex amplitude despite absence of a stable baseline. EMG from the obstructed arm was extrapolated from before the contact with the obstacle (−800–0 ms; linear regression) forwards in time beyond the contact with the obstacle. The actual EMG within the reflex analysis window (60–160 ms post contact) was then subtracted from this extrapolated signal within the same time window. This was performed for all Kohnstamm and Voluntary trials, and the mean stretch reflex amplitude was calculated in each participant. To determine whether a stretch response was present, a one sample *t*-test against 0 was performed in each condition. Differences across conditions were determined via a within subjects *t*-test.

We also investigated the detailed pattern of EMG during the obstacle phase at the level of single trials, to determine how afferent input from the obstacle affected the putative Kohnstamm generator. The previous linear trend analysis was insensitive to whether the EMG signal was truly flat during contact with the obstacle or just appeared that way due to averaging (see Supplementary Figure 2). We examined the first derivative of the rectified and smoothed EMG signal for both arms to quantify positive and negative signal change at the level of the individual trial (Julkunen et al., 2013). The positive and negative area under the curve (AUC) of the first derivative was calculated during several time windows for each individual trial, and divided by the duration of each window. The time windows of interest were: when the muscle was at rest (1000 ms window at start of the trial, prior to the induction and aftercontraction), immediately before contact with obstacle (500 ms window), during entire contact with the obstacle (~750 ms, first 250 ms excluded due to possible stretch responses), and immediately after release of the obstacle (500 ms window).

Signals from the strain gauge were analyzed to determine force perception and reproduction. The force with which the participant made contact with the obstacle was calculated by taking the amplitude of the first peak in the signal post-contact (**Figure 9B**). This was done to ensure the analysis matched the instruction for the participants to remember the initial contact force. Contact force was defined as the first peak in the signal from the strain gauge. We chose this approach to make our experiment commensurate with previous studies of sensory suppression which used discrete taps (Shergill et al., 2003). This was performed in four conditions: for all Kohnstamm trials, Voluntary trials and subsequent reproduction of forces on Kohnstamm and Voluntary trials. The mean contact force in each condition was analyzed with 2 × 2 within subjects ANOVA with the variables force type (initial force vs. force reproduction) and movement condition (Voluntary vs. Kohnstamm).

Video data was analyzed using ImageJ (Schneider et al., 2012) from 11 participants to determine: (1) angular displacement of the obstructed arm when it contacted the obstacle on Kohnstamm trials, Voluntary trials and force reproduction trials, (2) the maximum angle of both arms during Kohnstamm trials, and (3) effect of the obstacle on the angle of participant's trunk (posture). Data was lost for one participant due to recording equipment failure.

### Results

#### Effect of obstructing one arm on EMG in the other

During Kohnstamm, obstructing one arm caused EMG amplitude in that arm to change from its usual rising pattern (Figure 5) in the same manner as was seen in Experiment 1. However, there was no such change in the unobstructed arm. This manifested as a significant main effect of Arm [*F*_(1, 11)_ = 8.02, *p* = 0.02], a significant main effect of Time [*F*_(1, 11)_ = 12.88, *p* < 0.01] and a significant Arm x Time interaction [*F*_(1, 11)_ = 8.59, *p* = 0.01]. Planned comparisons revealed that during Kohnstamm the obstacle produced a significant change in the linear trend of the EMG signal from the obstructed arm [*t*_(11)_ = 4.04, *p* < 0.01]. There was no significant change in EMG acquired simultaneously from the unobstructed arm [*t*_(11)_ = 0.81, *p* = 0.43]. Trend values can be found in Supplementary Table 1.

#### EMG increases following obstacle removal

As can be seen from Figure 5, the removal of obstruction during Kohnstamm was accompanied by an immediate increase in the linear trend of EMG from the previously obstructed arm. ANOVA showed a significant Time × Arm interaction [*F*_(1, 11)_ = 6.09, *p* = 0.031], and no main effects of Arm or Time. Simple effects *t*-tests were used to investigate this interaction. We found that during Kohnstamm there was a significant increase in the linear trend of the obstructed arm EMG after release from the obstacle [*t*_(11)_ = −3.23, *p* < 0.01]. In contrast, *t*-tests revealed no significant effect of the obstacle release on the arm that was not blocked by the obstacle [*t*_(11)_ = 1.82, *p* = 0.096].

During Kohnstamm the EMG of the obstructed arm continued to increase after unblocking. There was no significant difference between the final EMG of the obstructed arm (mean = 1.11 mV, *SD* = 0.06 mV) and unobstructed arm [mean = 1.11 mV, *SD* = 0.06 mV; *t*_(11)_ = 0.48, *p* = 0.64]. Indeed, there was no significant difference between the maximum angular displacement of the obstructed arm (mean = 39.5°, *SD* = 19.76°) and unobstructed arm [mean = 39.83°, *SD* = 21.6°; *t*_(10)_ = 0.31, *p* = 0.76] on Kohnstamm trials.

#### Stretch reflex response is preserved during Kohnstamm

**Figure 6:**
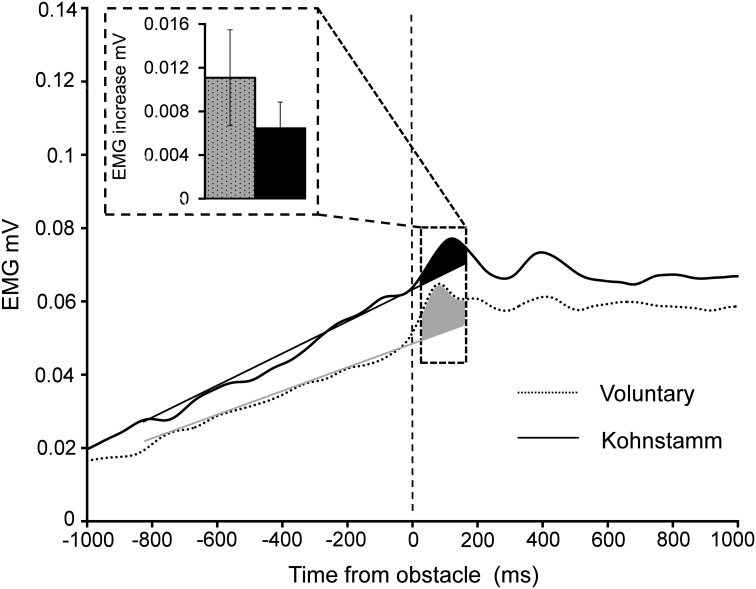
**Increase in EMG 60–160 ms post-contact with obstacle during Voluntary and Kohnstamm movements**. Insert shows the mean increase in EMG relative to a trend line fitted to pre-contact EMG on every trial. Trend line is shown for illustrative purposes.

A significant, transient increase in obstructed arm EMG (Figure 6) was found in *both* the Kohnstamm [*t*_(11)_ = 2.7, *p* = 0.02] and Voluntary movement [*t*_(11)_ = 2.52, *p* = 0.03] conditions after contacting the obstacle (60–160 ms post contact). However, the magnitude of this increase did not significantly differ *across* Kohnstamm and Voluntary movement conditions [*t*_(11)_ = −0.81, *p* = 0.43].

#### EMG during Kohnstamm obstruction: plateau or oscillation?

**Figure 7:**
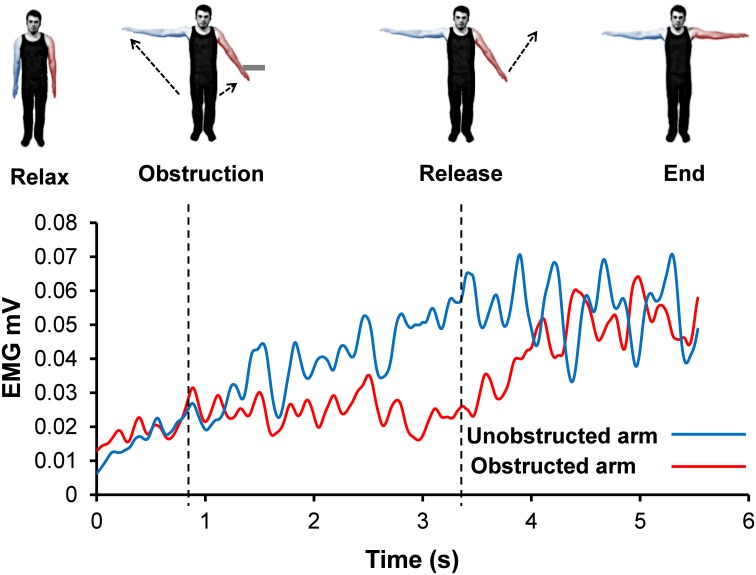
**Rectified and smoothed EMG from both arms from a single representative trial (illustrates the signal oscillation during contact with obstacle)**.

**Figure 8:**
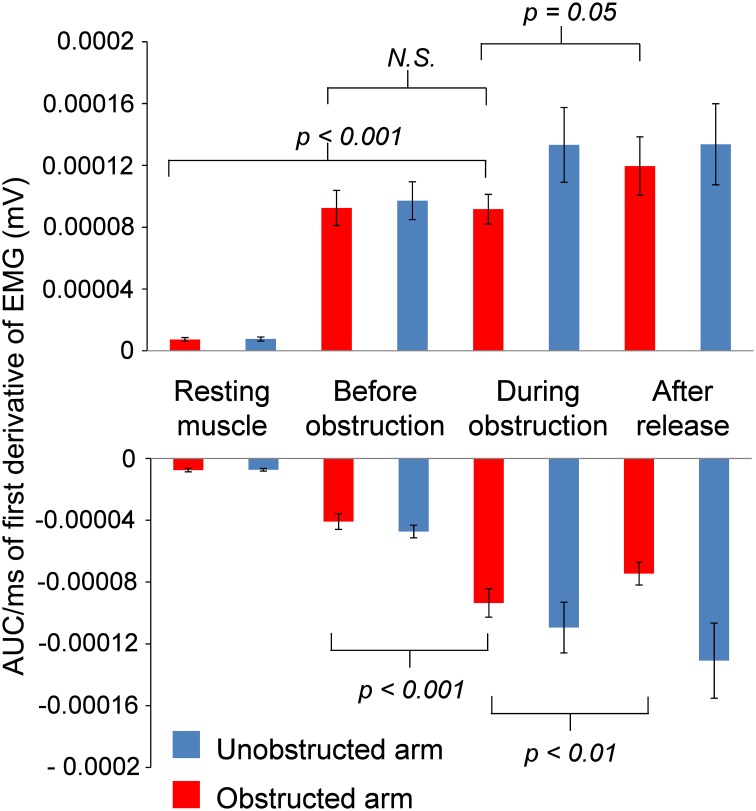
**Group average positive and negative AUC of first derivative of EMG for both Obstructed arm and Unobstructed arm**. Resting muscle refers to 1000 ms window at the start of the trial, before the Kohnstamm induction. Before obstruction refers to a 500 ms window immediately prior to contact with obstacle. During obstruction refers to a window that includes the entire time in contact with the obstacle (~1750 ms), excluding the first 250 ms (stretch response). After release is a 500 ms window immediately after obstacle has been removed. All AUC calculations are adjusted for the number of samples in each window.

Inspection of grand average EMG gives the impression that the EMG is flat during contact with the obstacle on Kohnstamm trials. However, inspection of individual trials suggested an oscillating pattern (Figure 7), with periodic increase and decrease of EMG throughout the obstacle contact phase. Because these oscillations were poorly time-locked to contact with the obstacle, they produced a flat EMG trace after averaging. To characterize this oscillatory pattern, we computed the signed positive and negative areas under the EMG first derivative (For further details see Supplementary Figure 2). On Kohnstamm trials both positive [*t*_(12)_ = 8.77, *p* < 0.001] and negative [*t*_(12)_ = 9.51, *p* < 0.001] EMG signal change were significantly higher during obstruction than when the muscle was at rest (Figure 8). Positive EMG signal change remained stable from before contact to during contact with the obstacle [*t*_(12)_ = 0.10, *p* = 0.92]. Contrastingly, negative signal change significantly increased [*t*_(12)_ = 6.48, *p* < 0.001] after obstruction compared to immediately before. This suggests strong downward adjustment in EMG triggered by contacting the obstacle. On Kohnstamm trials, when the arm is released from obstruction a significant reduction in negative signal change [*t*_(12)_ = 4.04, *p* < 0.01] and a trend toward increased positive signal change [*t*_(12)_ = 2.20, *p* = 0.05] was found, relative to during contact phase.

#### Kohnstamm forces are perceived as being stronger than voluntary forces

**Figure 9:**
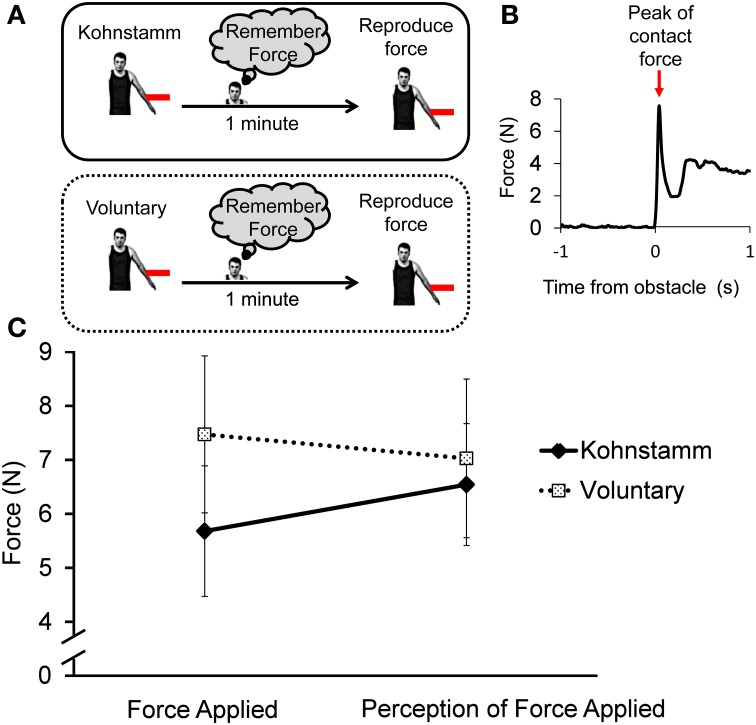
**Force of initial Kohnstamm and Voluntary movements and subsequent Voluntary reproductions after 1 min**. **(A)** In both conditions the movement generated a force and participant's had to remember the force and then reproduce it via a voluntary movement. **(B)** Force levels were defined based on the maximum amplitude of the first peak after contact with the stain gauge (shown is the initial force applied during a representative Kohnstamm trial). **(C)** There was significant interaction between force applied and the perception of that force across Kohnstamm and voluntary conditions [*F*_(1, 11)_ = 5.72, *p* = 0.04].

Figure 9A shows participants' attempts to voluntarily reproduce a Kohnstamm contact force. The reproductions were stronger than the initial Kohnstamm force [6.54 N (*SD* = 3.91) vs. 5.68 N (*SD* = 4.19)]. However, when asked to reproduce Voluntary forces, they reproduced weaker forces than the initial force [7.03 N (*SD* = 5.09) vs. 7.47 N (*SD* = 5.04)]. A 2 × 2 ANOVA with factors of movement condition (Voluntary, Kohnstamm) and force type (initial force, force reproduction) showed a significant Type × Condition interaction [*F*_(1, 11)_ = 5.72, *p* = 0.04; Figure 9C]. There was no main effect of force type [*F*_(1, 11)_ = 0.1, *p* = 0.76] or movement condition [*F*_(1, 11)_ = 2.04, *p* = 0.18]. *Post-hoc t*-tests to explore the interaction did not find any significant pairwise differences between conditions, showing that the interaction was based on a difference of differences.

It is possible that the differences between Kohnstamm and Voluntary trials may result from differences in arm position or body posture. For this reason video data from all trial types was examined. Mean angular displacement of the obstructed arm at contact with the obstacle did not differ between Kohnstamm trials (mean = 15.03°, *SD* = 4.3°), Voluntary trials (mean = 15.17°, *SD* = 4.14°), Kohnstamm reproduction trials (mean = 15.12°, *SD* = 4.48°) or Voluntary reproduction trials (mean = 15.96°, *SD* = 4.11°). Contact with the obstacle produced small but significant changes in the angle of the participant's trunk toward the obstacle. This was true for both Kohnstamm trials [mean = 0.76°, *SD* = 1.12°, *t*_(10)_ = 2.25, *p* < 0.05] and Voluntary trials [mean = 0.64°, *SD* = 0.86°, *t*_(10)_ = 2.47, *p* < 0.05]. However, there was no significant difference between the conditions.

### Discussion

During a bilateral Kohnstamm, unilateral obstruction resulted in a plateau of the obstructed arm EMG, but had no effect on the unobstructed arm EMG. This suggests there are separate Kohnstamm generators for each arm, and moreover that each generator processes its own arm-specific sensory feedback. Experiment 1 and previous studies (Forbes et al., 1926; Adamson and McDonagh, 2004) could not resolve whether sensory inputs permanently reset the output of the Kohnstamm generator to a new stable level, or merely temporarily gated the generator output while the obstacle was in place. The results of Experiment 2 clearly support the latter view. Removal of the obstacle caused the EMG signal to increase. Post-release EMG resumed the increasing trend seen prior to obstruction. Moreover, the obstructed arm reached a similar final level of EMG and angular displacement to the unobstructed arm.

EMG signals from the obstructed arm showed that contact with the obstacle produced an oscillating EMG pattern. Taking the first derivative of the EMG signal across the trial revealed that while obstruction is associated with an increase in negative signal change, positive signal change remained constant. These results show that the afferent input does not set the output of the Kohnstamm generator to a lower level. Rather, our data suggests that the generator continues to *specify* a steadily increasing EMG level. At the same time, afferent input associated with obstacle contact triggers an intermittent decrease in EMG. The combination of continuous, efferent-driven EMG increase and repeated, afferent-driven EMG decrease could explain the oscillating EGM patterns that we observed.

A significant, transient increase in EMG, consistent with a transcortical long loop reflex (Conrad and Meyer-Lohmann, 1980; Matthews, 1991), was found after both Kohnstamm and voluntary contact with the obstacle. This putative stretch response did not significantly differ in size between Kohnstamm and voluntary conditions, suggesting that the Kohnstamm induction does not alter the excitability of either the afferent spindle-driven or efferent arms of the long-loop reflex.

Finally our force reproduction task showed that Kohnstamm forces are perceived as stronger than equivalent voluntary forces. This is consistent with the possibility that Kohnstamm generators do not send an efference copy to the neural centers thought to underlie awareness of self-produced force (Blakemore et al., 1998; Shergill et al., 2003).

## General discussion

We physically obstructed the Kohnstamm aftercontraction by blocking arm movement with an obstacle. This resulted in a halt to the gradually increasing EMG signal that characterizes the Kohnstamm phenomenon. Experiment 1 found this for unimanual aftercontractions, where the occurrence of an obstruction was unpredictable. A similar result was found in Experiment 2 for bilateral aftercontractions, when the obstruction could be unpredictably supplied to either arm. Contact with the obstacle was associated with a transient stretch response in the activity of the muscle, which was similar in magnitude to that seen during matched voluntary movements. Removal of the obstacle caused the EMG signal to resume the characteristic increase found with aftercontractions. This increase resumed the linear trend seen prior to the introduction of an obstacle. Moreover, the obstructed arm reached a similar final level of EMG and angular displacement to the unobstructed arm, albeit with a 2 second delay due to the obstacle. Analysis of individual trials showed that the change in the EMG signal during obstruction was an oscillation with repeated negative corrections preventing the gradual rise of EMG that characterized the Kohnstamm. During bilateral aftercontractions, the unobstructed arm was unaffected by the obstacle applied to the other arm. In both experiments Kohnstamm forces were overestimated relative to voluntary forces.

### Central models of Kohnstamm generation

Purely ballistic, central feedforward models of the Kohnstamm phenomenon have been proposed based on persistence of the inducing voluntary motor command (Salmon, 1914, 1916) or cortical excitation (Sapirstein et al., 1936, 1938). These purely central models seem inconsistent with our finding of afferent-triggered changes in EMG.

### Peripheral models of Kohnstamm generation

The Kohnstamm drive could come entirely from peripheral signals. On this view, the induction phase would lead to some change in a peripheral signal that drives motor circuits. One model views the Kohnstamm phenomenon as a form of proportional-integral-derivative (PID) control, similar to equilibrium point control (Feldman, 1986; Bizzi et al., 1992), proposed for both stretch reflexes and voluntary actions. For such control, a central motor signal setting the equilibrium point of the muscle would result in a follow-up servo contraction of the muscle, causing a movement toward that position. However, in these simple, linear equilibrium-point models, the EMG signal would be greatest at the start of the movement, when the muscle is far from its desired length, and would then decrease. In fact, we found that EMG increases as the arm moves, consistent with previous reports.

Alternatively, the equilibrium point might move gradually over time, defining a virtual trajectory (Bizzi et al., 1984; Hogan, 1985). On these models, the EMG level after release of an obstacle should be higher than before the obstacle was applied, and higher than the EMG level at the same point on unobstructed trials. The equilibrium point would shift farther ahead of the actual limb position during any period of obstruction, leading to an increased force on release. This pattern was not found in our data.

One influential peripheral account holds that spindle response properties are altered following prolonged isometric contraction during the induction phase (Hagbarth and Nordin, 1998). On this view, Kohnstamm induction might cause a high number of stable cross-bridges to form between actin and myosin in intrafusal fibers. The persistence of these cross-bridges maintains stiffness in the intrafusal fibers leading to excitation of primary spindle endings (Proske et al., 1993), which in turn feeds back to motor regions causing the EMG to increase (Gregory et al., 1988; Hagbarth and Nordin, 1998; Duclos et al., 2004).

Indeed, it has been reported that such muscle thixotropy leads to a shift in the perceived position of the elbow joint in the same direction as a previous isometric contraction (Tsay et al., 2014). Perhaps a combination of this sensory change and equilibrium point control explains the Kohnstamm phenomenon. The thixotropy account predicts that Kohnstamm induction should produce a *perceptual* illusion of the shoulder being abducted. However, to produce a *movement* of the shoulder, the equilibrium point of the muscle must also shift, and by an amount greater than the altered sensory signal. The equilibrium point account has been discussed above. However, the experience of the Kohnstamm seems less like a perceptual illusion of position sense than a veridical perception of an unexplained movement. Indeed, previous studies suggest that position sense is normal during Kohnstamm phenomenon (Howard and Anstis, 1974). In addition, we have shown the equilibrium point accounts cannot readily explain the full features of the Kohnstamm EMG pattern. It therefore remains unclear whether such peripheral mechanisms can fully account for the Kohnstamm phenomenon.

We attempted to measure the transient stretch response due to obstruction during the Kohnstamm phenomenon, apparently for the first time. The timescale of the stretch response was comparable to the transcortical long loop reflex (Conrad and Meyer-Lohmann, 1980; Matthews, 1991). Existing peripheral accounts of the Kohnstamm phenomenon posit high spindle sensitivity and/or increased spindle discharge (Gregory et al., 1988; Hagbarth and Nordin, 1998; Duclos et al., 2004) during the aftercontraction. We found that the stretch response was actually slightly (though non-significantly) *smaller* on Kohnstamm movements compared to matched voluntary movements. The state of the muscle spindles in both our Kohnstamm and voluntary movement conditions could not be measured directly. However, our results seem incompatible with peripheral accounts of the Kohnstamm phenomenon that are based on increased excitability.

### Hybrid models

Our data supports previous claims that both central and peripheral signals contribute to aftercontractions. Our results show that sensory feedback can modulate the putative Kohnstamm generator, but that some aspects of the drive remain independent of sensory input (Parkinson and McDonagh, 2006). Obstructing a movement, as in our data, would trigger simultaneous afferent signals from muscle spindle, skin and tendon receptors, *inter alia*. One model gives force sensing, perhaps from Golgi tendon organs, a key role in the Kohnstamm, by suggesting a positive feedback loop between muscle force and Kohnstamm generator (Parkinson and McDonagh, 2006). However, the effects of release from obstruction seem inconsistent with this model. When an obstacle is removed, there is a sudden decrease in the load on the muscle, (Marsden et al., 1976), causing a decrease in tendon organ firing. A positive force feedback model would therefore predict a decrease in EMG, at least transiently. Instead, we observed an immediate *increase* in EMG following muscle unloading, and a return to the preceding EMG pattern. We suggest that the immediate resumption of EMG increase on obstacle release must reflect a persistent central drive from the Kohnstamm generator, rather than a feedback loop involving the periphery.

**Figure 10:**
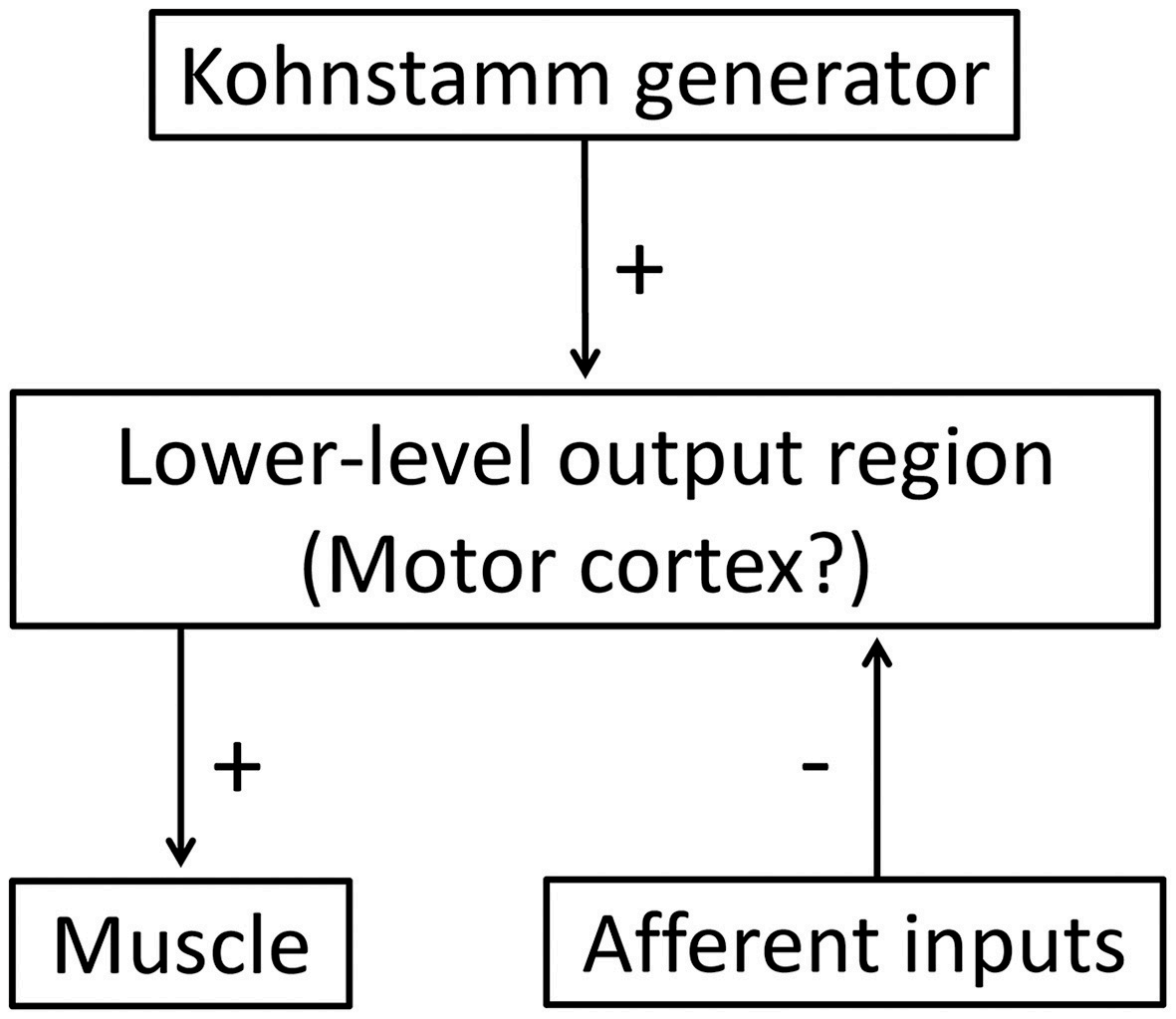
**A hybrid model of the Kohnstamm circuit**. Note that afferent input has a suppressive effect on the motor commands output from the lower-level motor region, but there is no afferent feedback to the generator itself. See text for further details.

Some models have suggested that Kohnstamm induction causes central excitatory changes within the brain regions responsible for generating muscle tone, and that these changes decay over time (Craske and Craske, 1986; Ghafouri et al., 1998; Gurfinkel et al., 2006). Thus, the “normal” role of the Kohnstamm generator would be to provide output that achieves and maintains stable muscle lengths, and thus body posture (Fessard and Tournay, 1949; Gurfinkel et al., 1989; Ghafouri et al., 1998; Adamson and McDonagh, 2004; Duclos et al., 2004). Postural control requires peripheral input and central compensatory commands to achieve the desired posture in response to changes in the environment (Cordo and Nashner, 1982). Since the processes for *maintaining* current posture are generally slow and sustained, it follows that the system would return to an underlying pattern of motor output once the afferent input returned to normal levels. This is consistent with the pattern of results we observed, and with a hybrid model of the Kohnstamm phenomenon. In the case of the Kohnstamm phenomenon, output from the generator is much higher than normal, due to the induction period. The present results indicate that the output from this generator can be gated by afferent signals. We observed that, at the level of individual trials, the EMG signal shows an oscillation during contact with the obstacle. The EMG continually increases, but is then repeatedly reset to a lower level while contact continues. This produces a reduced mean level of activity over time. When contact with the obstacle is ended, the gate is reopened, and EMG again rises. We found that the EMG and angular displacement of the obstructed arm reached the same final levels as the unobstructed arm. EMG increase after obstacle removal was also much more rapid than the 1–3 s it takes for the aftercontraction to begin after the relaxation of the arm (Csiky, 1915; Pinkhof, 1922). These findings indicate that the Kohnstamm generator is not suspended during obstacle contact. Rather, it continues to generate motor commands, but these commands are repeatedly corrected by a circuit driven by afferent input. This could be achieved by a high level generator outputting to a low-level sensorimotor control circuit, which in turn outputs to the muscle. Afferent input would have a suppressive effect on this lower-level circuit, but no effect on the highest level command generator (Figure 10). Interestingly, two studies reported that voluntary movements immediately after the induction could reduce aftercontractions (Hutton et al., 1987; Duclos et al., 2004), suggesting that the sensorimotor processes underlying the Kohnstamm movement can be partly reset by voluntary commands.

### Laterality

Our results indicate independence of the Kohnstamm generators that control each arm. Obstructing one arm after a bilateral induction did not significantly affect the Kohnstamm phenomenon in the other arm. Theoretically, this could also be achieved by a single generator outputting to separate lower level areas, which receive separate afferent input. Nevertheless, this unilateral organization suggests that the EMG effects seen in Experiment 1 and 2 are unlikely to reflect a voluntary reaction to contacting the obstacle. Voluntary reactions, particularly fast inhibitory reactions, are generally organized bilaterally (Coxon et al., 2007; Garbarini et al., 2012; Mattia et al., 2012). Previous functional magnetic resonance imaging (fMRI) studies have found wide ranging bilateral activity in sensorimotor and cerebellar regions during Kohnstamm aftercontraction (Duclos et al., 2007; Parkinson et al., 2009). This suggested that the Kohnstamm generators are not completely separate. However, these inferences are based on correlational neuroimaging data, and cannot distinguish between the generator itself and correlated epiphenomenal activations. Our results indicate that ipsilateral brain activations in these studies may not be output from the Kohnstamm generator to the muscle. Instead it could reflect normal sensorimotor feedback, or some epiphenomenal activation. Previous studies of bilateral aftercontractions reported that the pattern of oscillation in one arm influenced the other (Craske and Craske, 1986), just as in bimanual voluntary movements. Further, proprioceptive input from the ipsilateral arm can influence the velocity of a contralateral aftercontraction (Brun et al., 2015). Further work is required to characterize the effect of contralateral input on the Kohnstamm movement.

### Subjective experience during the Kohnstamm phenomenon

Voluntary and involuntary movement may be physically identical, yet subjectively feel very different. The enduring scientific interest in the Kohnstamm phenomenon may relate to the strange feelings it produces (Forbes et al., 1926; Craske and Craske, 1985). Like other examples of “voluntariness” and “involuntariness,” these experiences often elude experimental measurement. We developed novel, quantitative and implicit measures of subjective experience during Kohnstamm phenomena, based on the perceived contact force when movement encounters an obstacle. We found that Kohnstamm forces were overestimated relative to voluntary forces in both experiments. This overestimation of Kohnstamm forces is consistent with the view that the Kohnstamm generator does not send the efference copies used to cancel against the sensory consequences of the action (Blakemore et al., 1998). The precise origin of efference copies remains controversial. However, several studies suggest efference copies underlying perceptual attenuation of self-generated events originate at a relatively high level of the action control hierarchy, upstream of the primary motor cortex (Haggard and Whitford, 2004; Voss et al., 2006). Neuroimaging studies of the Kohnstamm phenomenon showed activation in primary motor areas during aftercontractions (Duclos et al., 2007), but, interestingly, did not show significant activations of the medial frontal regions hypothesized to generate the efferent signals that contribute to action awareness (Fried et al., 1991; Haggard and Magno, 1999; Haggard and Whitford, 2004; Haggard, 2011).

Lack of efference copy might suggest that the Kohnstamm phenomenon should feel similar to passive movements. However, Kohnstamm and passive movements are easily distinguishable. In fact, our participants rated Kohnstamm forces as being stronger than passive movements in Experiment 1, though this result should be interpreted with caution, as we were unable to precisely match the force ranges for the two conditions. In addition, the sensory signals reaching the brain differ profoundly between passive movement and the Kohnstamm phenomenon. In passive movement, there is a strong additional external input not present in the Kohnstamm case, from the experimenter's handling of the participant's passive arm. It remains to be determined whether the other reported phenomena associated with Kohnstamm movement, such as the lightness of the arm, result from the absence of these additional inputs or some other more fundamental difference between passive and Kohnstamm movement.

## Conclusion

In conclusion, the Kohnstamm phenomenon is modulated by peripheral sensory input. Our results are not consistent with the view that the Kohnstamm generator is a simple PID controller, in which a single peripheral signal, such as muscle position or force is driven to a target level by a sensory feedback loop. Rather, the Kohnstamm phenomenon depends on an apparently central generator, whose output is temporarily gated, or limited by the sensory signals produced during contact with the obstacle. Further, the Kohnstamm generator is hemispherically lateralized, and presumably located contralateral to the moving limb. The Kohnstamm generator appears not to transmit efference copies to the brain centers responsible for action awareness, thus explaining some of the strange sensations associated with the phenomenon. Our results fit within a framework that views the Kohnstamm phenomenon as a by-product of adaptations within a complex postural control system. In particular, postural control often requires motor drive to be maintained over long periods while cognitive control capacity is directed toward other tasks. Interestingly, this drive can persist when the peripheral environment changes. Our results also shed important light on the nature of voluntary and involuntary movement control. We show that movements that are involuntary can nevertheless be well-organized, persistent, and environment-sensitive. Despite all the sophisticated information-processing that modulates Kohnstamm after-contractions, they nevertheless *feel* completely different from voluntary actions. Our results highlight that awareness of action involves a complex interplay between central commands and peripheral signals. The interactions between these signals may occur at multiple levels of the motor hierarchy. Most importantly, our results suggest that some specific central generator circuits produce an experience of voluntariness, while others, like the Kohnstamm generator, do not—irrespective of the specific peripheral circuits they engage. Future research might usefully focus on identifying those key features that cause some central motor generators, but not others, to trigger an experience of voluntariness.

### Conflict of interest statement

The authors declare that the research was conducted in the absence of any commercial or financial relationships that could be construed as a potential conflict of interest.
