# Supplementary material for: Sensorimotor organization of a sustained involuntary movement
Source: Front Behav Neurosci. 2015 Jul 28;9:185. doi: 10.3389/fnbeh.2015.00185 (PMC4517064; doi:10.3389/fnbeh.2015.00185)
Supplement: Supplementary file 1 [file DataSheet1.PDF]

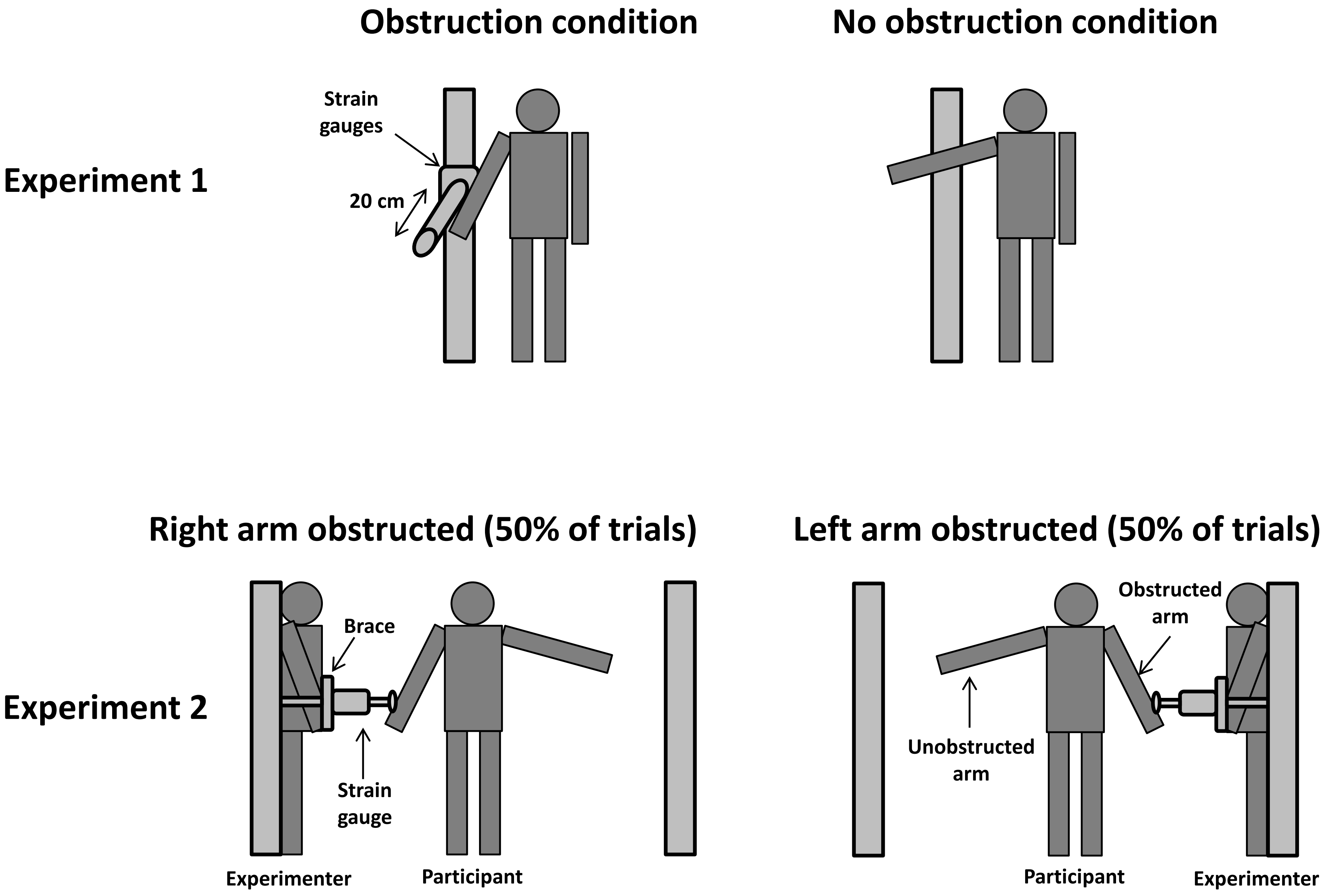

Supplementary Figure 1. *Diagram showing how obstruction was applied in Experiment 1 and 2. In Experiment 1 the obstacle was in a fixed location that was determined individually for each participant at the start of the session. On 'Obstruction trials' participants stepped forward from the wall used for the induction and the arm was stopped by the obstacle as it rose. Strain gauges were mounted at the base to record the force with which they were abducting against the obstacle. On 'No obstruction' trials the obstacle was removed by the experimenter without the participant's awareness. On these trials the arm could freely rise. In Experiment 2 obstruction was applied directly to one of the arms. This was randomised and participants were not aware which arm would be obstructed. The obstacle was attached directly to a single strain gauge. The strain gauge was attached to a brace. The experimenter stood such that the brace was contacting a solid surface and the force of the abduction could be recorded. The other arm could rise freely. After ~2 seconds the experimenter removed the obstruction, allowing the obstructed arm to also rise.*

Experiment	Condition/Arm	Trend before obstacle contact	Trend after obstacle contact	Trend before obstacle release	Trend after obstacle release
Exp. 1	Obstruction	0.0250 (0.0236)	-0.00102 (0.00763)	NA	NA
	No obstruction	0.0167 (0.0169)	0.0193 (0.0169)	NA	NA
Exp. 2	Obstructed arm	0.0283 (0.0193)	-0.00505 (0.0136)	-0.00292 (0.00495)	0.01978 (0.0221)
	Unobstructed arm	0.0274 (0.0192)	0.0226 (0.0283)	0.0208 (0.0338)	-0.00204 (0.0157)

Supplementary Table 1. *Table showing mean and standard deviation (in parentheses) of trend values of EMG signals. Values are shown for both experiments. For experiment 1, values are shown for the Obstruction and No obstruction conditions for the 1 second before contact with the obstacle and 1 second after obstacle contact. For experiment 2, values are shown for the Obstructed arm and Unobstructed arm for the 1 second before contact with the obstacle and 1 second after obstacle contact and for the 1 second before obstacle release and 1 second after obstacle release. Trend values were calculated by multiplying EMG in four 250ms bins by standard coefficients (*-3, -1, 1, 3*) for each participant. The mean of these four values was then calculated to give the trend values for each participant in each 1 second window of interest.*

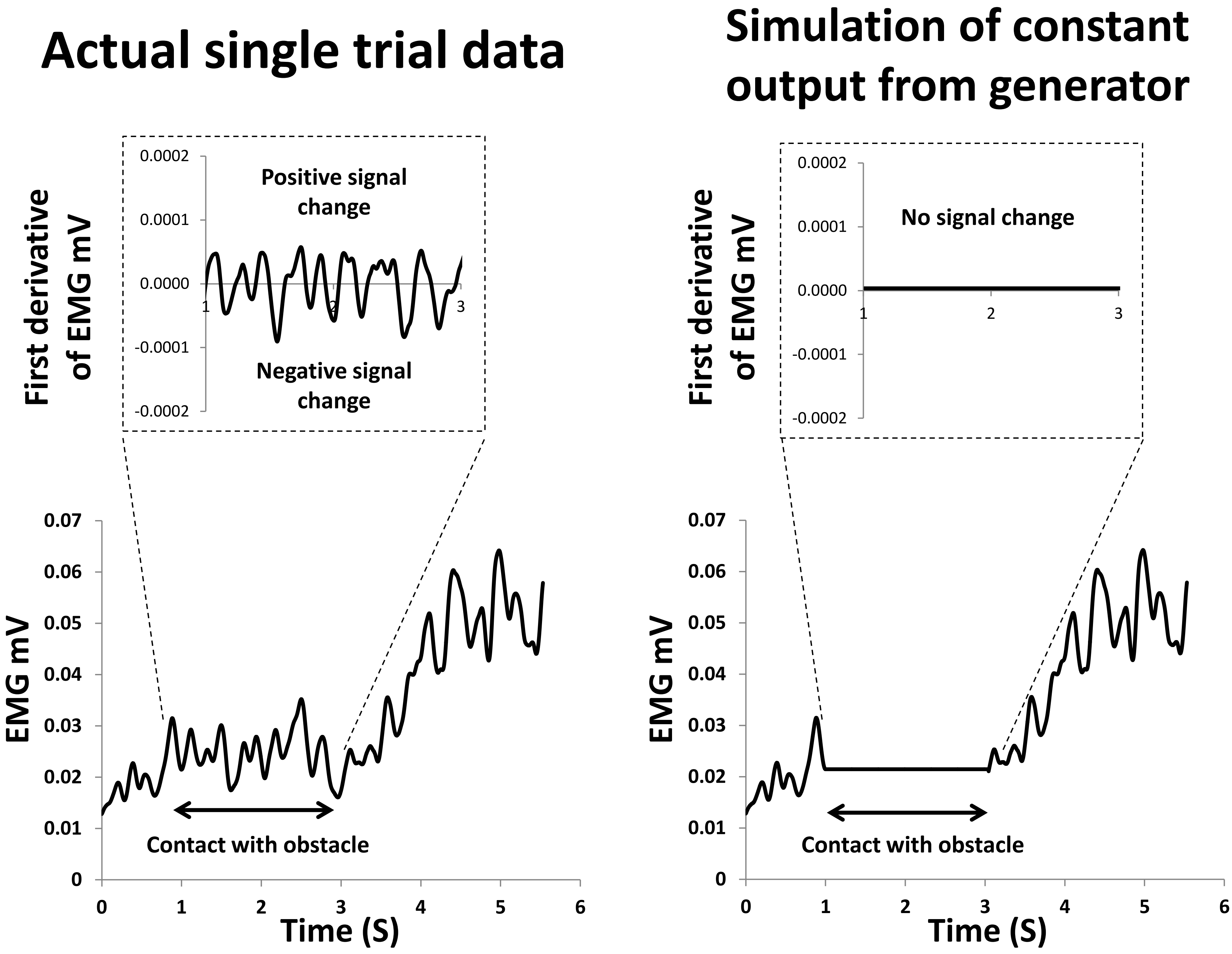

Supplementary Figure 2. *Actual and simulated EMG from the obstructed arm during a single Kohnstamm trial in Experiment 2. Simulated data (shown on the right) is what could be expected if the obstacle caused the EMG to plateau at the level of a single trial. In this case, taking the positive and negative AUC of the first derivative of the EMG signal would show that there was no signal change during obstruction. If this was observed, one might conclude that afferent input was resetting the output of the Kohnstamm generator to a new temporary and constant value. However, the actual data shown on the left is representative of what was found at the group level. By calculating the positive and negative signal change for each trial it was possible to show that the Kohnstamm generator does not take on a new constant output value. We could also separately compare positive and negative signal change during obstruction to signal changes immediately before and after obstruction. This facilitated a better characterisation of the effect of afferent input on the Kohnstamm generator.*
